# Smartphone addiction is more harmful to adolescents than Internet gaming disorder: Divergence in the impact of parenting styles

**DOI:** 10.3389/fpsyg.2022.1044190

**Published:** 2022-12-14

**Authors:** Zhao-kang Li, Li-juan Shi, Xin-lu Cai

**Affiliations:** ^1^School of Education, Hunan University of Science and Technology, Xiangtan, Hunan, China; ^2^Department of Physiology, Institute of Brain Science, School of Basic Medical Sciences, Hangzhou Normal University, Hangzhou, Zhejiang, China

**Keywords:** smartphone addiction, Internet gaming disorder, adolescent, mental health, parenting styles

## Abstract

**Background:**

The adverse effects of smartphone addiction (SPA) and Internet gaming disorder (IGD) on adolescents’ mental health have been widely recognized. However, the influence of parenting styles on these high-risk Internet use behaviors of adolescents still remain elusive. Aiming to identify preventable patterns for adolescents with SPA or IGD, this study compared the mental health status between adolescents with SPA and IGD and used path analysis to confirm actual effects of parenting styles on SPA and IGD.

**Methods:**

Participants were enrolled at a junior high school in Hunan Province and a senior high school in Shanxi Province, China [*n* = 3,049, female (male): 50.5% (49.5%), mean age = 15.68 ± 1.54]. All participants reported their socio-demographic characteristics and undertook standardized assessments of SPA, IGD, parenting styles, depression, anxiety, insomnia, self-control, and support utilization.

**Results:**

High levels of parental care and low levels of parental overprotection benefited adolescents’ mental health with SPA and IGD. However, despite having a more positive parenting style, adolescents with only SPA showed more severe mental health problems than adolescents with only IGD. Furthermore, the results showed that the parenting style of encouraging autonomy might be a protective factor against IGD, but it might reinforce SPA indirectly by reducing abilities of support utilization and self-control in whole sample.

**Conclusion:**

Compared to IGD, SPA which included different kinds of Internet addiction behaviors, was more hazardous for adolescents’ mental health. The divergent effects of an autonomy-encouraging parenting style on SPA and IGD may reflect the different impacts of self-control in different types of Internet addiction.

## Introduction

Internet gaming disorder (IGD), defined as problematic and impulsive use of Internet-based games, has a significant impact on physical and mental health (Kuss et al., 2014; van Rooij et al., 2014). IGD is associated with depression (Yen et al., 2019), anxiety (Bonnaire and Baptista, 2019), social difficulties (Lo et al., 2005), attention deficits (Swing et al., 2010), and poorer sleep quality (Sarda et al., 2016). Meanwhile, as a new portable device, the smartphone has opened a new era of mobile Internet (Cho, 2015), which leads more and more people becoming over-dependent on smartphones (Kwon et al., 2013). Researchers have proposed that the behavior of excessive use of smartphones can be characterized as smartphone addiction (SPA) (Pavia et al., 2016; Panova and Carbonell, 2018). Previous studies have also demonstrated that SPA is highly associated with depression and anxiety (Elhai et al., 2017; Elhai and Contractor, 2018; Pancani et al., 2020), emotional dysregulation (Rozgonjuk and Elhai, 2019), social deficits (Hawi and Samaha, 2017), and decreased sleep quality (Orzech et al., 2016). Importantly, IGD is different from SPA. For example, Petry et al. (2014) suggested that Internet gaming should be distinguished from other online activities. Moreover, smartphones are not solely carriers of gaming but a carrier of various online activities (e.g., socializing and shopping) (Montag et al., 2015a). Therefore, SPA is a concentrated manifestation of Internet addictive behaviors and may have disparate impacts on mental health compared with IGD. However, few studies have directly compared the effects of IGD and SPA on mental health in adolescents. Importantly, depression is regarded as the most severe psychological illness for individuals under 25 years old (Gore et al., 2011), which is usually accompanied by anxiety (Ko et al., 2012). Additionally, sleep quality has a remarkable impact on the physical and mental development of adolescents (Adams et al., 2013; Brand et al., 2014). Thus, this study aims to compare depression, anxiety, and insomnia between adolescents with IGD and adolescents with SPA.

Parenting style, as one of the vital family factors, has a critical impact on the development of adolescents. Furthermore, parenting styles closely relate to addictive behaviors in adolescents (Miller and Plant, 2010). Previous studies have shown that positive parenting styles (e.g., a caring parenting style) decrease smartphone dependence (Lian et al., 2016) and reduce the probability of IGD in adolescents (Floros et al., 2013). Meanwhile, negative parenting styles (e.g., an overprotective parenting style) increase smartphone dependence (Bae, 2015) and can result in IGD (Huang et al., 2010). However, the effects of encouraging autonomy (encouraging children to be independent and autonomous) on IGD and SPA remain elusive. Researchers have shown that parental encouragement of autonomy is associated with higher levels of well-being and fewer developments of behavioral problems in children (Jungert et al., 2015), implying that encouraging autonomy may be as beneficial as caring in managing adolescents’ high-risk Internet use behaviors. For example, a study on adolescents in Hong Kong showed that restricting adolescents’ Internet use increased their probability of Internet addiction (IA) by 1.9 times (Wu C. S. T. et al., 2016), supporting the suggestion of giving adolescents freedom to use the Internet. However, a study based on Korean elementary schoolchildren found that a lack of restrictions on using the Internet was related to IA among boys (Lee and Ogbolu, 2018), indicating the importance of supervision over children’s Internet use.

In addition, self-control is a protective factor against IGD (Kim et al., 2008) and SPA for adolescents (Gökçearslan et al., 2016). Self-control is a limited resource, and individuals with more self-control resources can more easily inhibit, change, or maintain activities to achieve their desired goals (Muraven and Baumeister, 2000). Dysfunctional self-control has been proposed as a core feature of IGD (Spada, 2014) and SPA (Kim et al., 2016). Critically, self-control is affected by parenting styles (Crosswhite and Kerpelman, 2012). Early experiences of parental acceptance (e.g., positive parenting styles) or rejection (e.g., negative parenting styles) have persistent effects on the development of self-control abilities in children (Hagger et al., 2010). Previous studies indicated that parenting styles are likely to influence IGD or SPA indirectly by altering self-control resources. Moreover, parenting styles also impact individuals’ ability to utilize social support (de Vries et al., 2016). Social support utilization refers that an individual could integrate into his or her social system and accept support which helps their physical and psychological development in interactions with system members (e.g., friends and teachers) (Brown and Larson, 2009). With a stronger ability to use social support, an individual can be regulated by their social system, which can complement their self-control resources and reduce undesirable behaviors (Hirschi, 1977; Baker, 2010; Wright et al., 2010). Thus, the utilization of social support, influenced by parenting styles, affects the self-control of adolescents. More importantly, social support is a predictive variable for IGD (Young et al., 2012) and SPA (Mo et al., 2018) as well. Researchers proposed that when a third variable transmits the effect of one variable to another, it may play the mediation role between these two variables (MacKinnon, 2008). When there are more than a single variable that can transmit effects between those two variables, these variables may play the role of multiple mediation (Hayes, 2009). Therefore, the present study explores the potential multiple mediation roles of support utilization and self-control between parenting styles and IGD or SPA.

An immature brain and poor self-control (Hong et al., 2013)can easily lead adolescents falling into trouble with IGD (Spada, 2014) and SPA (Lopez-Fernandez et al., 2014), resulting in an increased risk of depression, anxiety, and insomnia in adolescents. Therefore, it is of great significance to explore the pathways of parenting styles’ influences on IGD and SPA, which can guide coping with these issues in adolescents. This study was conducted based on the following hypotheses. H1: There are significant differences in depression, anxiety, and insomnia between adolescents with IGD and adolescents with SPA. H2: Positive parenting styles negatively predict IGD and SPA, and negative parenting styles positively predict IGD and SPA. H3: Support utilization and self-control play multiple mediation roles in the relationship between parenting style and IGD or SPA.

## Materials and methods

### Participants

The questionnaires were distributed to a junior high school in Hunan Province and a senior high school in Shanxi Province, China, from March to April 2021 in the form of paper questionnaires and online questionnaires on the Wenjuanxing website.^1^ After excluding participants with missing or omitted entries on the relevant scales and potentially irresponsible completion, the subsequent analysis included 3,049 samples. The overall sample consisted of 1,508 male subjects (49.5%) and 1,541 female subjects (50.5%), with a mean age of 15.68 years (SD = 1.54, age range 11–19) (Table 1).

**TABLE 1:** Socio-demographic characteristics of junior and senior high school students.

Characteristic	Number	Percentage (%)
**Gender**		
Male Female	1,508 1,541	49.5 50.5
**Age**		
11–13 14–16 17–19	321 1,877 851	10.53 61.56 27.91
**Being the only child**		
Yes No	514 2,535	16.9 83.1
**Living area**		
Urban Rural	1,257 1,792	41.2 58.8
**Education of mother**		
Lower than high school High school or equivalent Higher than high school	2,841 145 63	93.2 4.8 2.0
**Education of father**		
Lower than high school High school or equivalent Higher than high school	2,776 187 86	91.0 6.10 2.90
**Family socio-economic status**		
1–3 4–6 ≥7	448 2,042 559	14.7 67.0 18.3
**Survey method**		
Paper-based survey Online survey	1,490 1,559	48.9 51.1

The family socio-economic status were rated on a 10-point scale, with 1 indicating “very low degree of family income and social status” and 10 indicating “very high degree of family income and social status”.

### Measures

We used a multiple-choice question format to survey participants’ smartphone usage to indicate potential types of Internet addiction behaviors for SPA (Supplementary Table 1). Socio-demographic characteristics were collected, including gender, age, whether they were an only child, living area, the parents’ education degree, and the family socio-economic status degree.

#### Mobile phone addiction index (MPAI)

The MPAI scale measures the smartphone addiction degree of adolescents (Leung, 2008). The scale consists of 17 items. All items use a five-point Likert scale from 1 (never) to 5 (always). Total scores above or equal to 51 are considered smartphone addiction, with higher scores indicating a more severe level of smartphone addiction (Zhang et al., 2019). In this study, Cronbach’s alpha coefficient of the scale was 0.907.

#### Internet gaming disorder scale (IGD)

The short-form IGD scale measures whether subjects meet the Diagnostic and Statistical Manual of Mental Disorders-5 (DSM-5) criteria for Internet gaming disorder (Sigerson et al., 2017). The scale consists of nine items scored 0 or 1 (yes = 1, no = 0), with total scores ranging from 0 to 9. In the present study, Cronbach’s alpha coefficient of the scale was 0.861.

#### Parental bonding instrument (PBI)

The PBI measures the parenting styles that adolescents experience (Kazarian et al., 1987). The Chinese version of the father’s and the mother’s PBI contains 23 items for each, including the caring dimension (referring to parents’ gentleness, understanding, and support for their children), the encouraging autonomy dimension (referring to parents’ encouragement of their children’s independence), and the overprotection dimension (referring to parents’ strict restriction of their children’s freedom) (Chu et al., 2009). In the present study, Cronbach’s alpha coefficients for each dimension were 0.798 (mother’s care), 0.875 (mother’s encouragement of autonomy), 0.836 (mother’s overprotection), 0.789 (father’s care), 0.884 (father’s encouragement of autonomy), and 0.883 (father’s overprotection).

#### Generalized anxiety disorder-7 (GAD-7)

The GAD-7 scale screens for anxiety symptoms (Yu et al., 2016). The scale consists of seven items, and all items use a 4-point scale. The total score ranges from 0 to 21, with higher scores indicating higher degrees of anxiety. In the present study, the Cronbach alpha coefficient was 0.927.

#### Patient health questionnaire-9 (PHQ-9)

The PHQ-9 scale screens for depressive symptoms (Wang et al., 2014). The scale has nine items, and all items use a 4-point scale. The total score ranges from 0 to 27, with higher scores indicating more severe depressive symptoms. In the present study, Cronbach’s alpha coefficient was 0.905.

#### Athens insomnia scale (AIS)

The AIS screens for sleep disturbances (Chung et al., 2011). The scale has eight items and uses a 4-point scale. The total score ranges from 0 to 24, with higher scores indicating more severe sleep disturbances. In this study, Cronbach’s alpha coefficient of the scale was 0.844.

#### Self-control scale (SCS)

The SCS measures the self-control ability of adolescents (Tangney et al., 2004). The Chinese version consists of 19 items rated on a 5-point scale, with 1 indicating ere sleep disturbances. In this study, ing more severe depressive symptoms. In thehe better the self-control ability (Tan and Guo, 2008). In this study, Cronbach’s alpha coefficient of the scale was 0.863.

#### Social support rating scale (SSRS)

This SSRS includes 17 items of subjective support, objective support, and utilization of support in three dimensions (Wu X. S. et al., 2016). A 5-point scale was used, with 1 indicating rt, objective support, and 1 indicating ere sleep disturbances. In this study, ing moreutilization dimension indicate better use of social support. Cronbach’s alpha coefficient for the support utilization dimension was 0.921.

### Statistical analysis

According to the criteria of the Chinese version of MPAI, 807 individuals (26.50% of the overall participants) had SPA in this study (MPAI total scores ≥51) (Zhang et al., 2019). This proportion matched the SPA proportions previously investigated in Chinese (21.3%) (Long et al., 2016) and Asian (14.0n matched Sohn et al., 2019) studies with the same age sample. Thus, we divided all participants into the No SPA group (<51) and SPA group (≥51) based on the total MPAI score. A total IGD score ≥6 was used to for the IGD group (Lemmens et al., 2015). Subsequently, all participants were divided into the No SPA-No IGD group (*n* = 2,002), SPA-IGD group (*n* = 257), Only IGD group (*n* = 240), and Only SPA group (*n* = 550). Then, the following analyses were performed. (1) ANOVA was used to compare the differences in depression, anxiety, insomnia, self-control, support utilization ability, and parenting styles among the four groups. (2) A multiple regression analysis was conducted to identify the factors that influence depression, anxiety and insomnia. (3) Relative weight analysis was conducted to diagnose the factors that contribute most to depression, anxiety and insomnia. (4) Another multiple regression analysis was conducted to single out parenting styles that influence SPA or IGD. (5) After controlling for gender and age, we used the maximum likelihood (ML) method to construct path models to test the multiple mediating roles of support utilization and self-control in the relationship between parenting styles and SPA or IGD. All statistical analyses were performed in SPSS 22.0, R 4.2.0 and Mplus 7.2. Estimation equations for multiple regression analysis, relative weight analysis, and multiple mediation analysis are detailed in Supplementary Table 2.

## Results

### Differences in psychological variables among the four groups

There were significant differences among the four groups in depression, anxiety, insomnia, self-control, and support utilization.

*Post-hoc* tests showed that depression and anxiety levels increased sequentially among the groups (No SPA-No IGD group < Only IGD group < Only SPA group < SPA-IGD group). While the insomnia levels increased sequentially among the groups [No SPA-No IGD group < Only IGD group < Only SPA (SPA-IGD) group], there was no significant difference between the SPA-IGD and Only SPA groups. Similarly, although the self-control and support utilization levels decreased sequentially among the groups [No SPA-No IGD (Only IGD) group > Only SPA group > SPA-IGD group], there was no significant difference between the No SPA-No IGD group and the Only IGD group (Table 2).

**TABLE 2:** Differences in psychological variables among the four groups (*n* = 3,049).

Psychological variables	Groups	M ± SD	*F*	η*p*^2^	*P*	95% CI	*Post-hoc* test
Depression	1.No SPA-No IGD	6.52 ± 5.18	140.91	0.122	<0.001	(0.10, 0.14)	1 < 2 < 3 < 4
	2.Only IGD	7.94 ± 5.90					
	3.Only SPA	10.44 ± 6.09					
	4.SPA-IGD	12.58 ± 6.58					
Anxiety	1.No SPA-No IGD	4.49 ± 4.51	121.64	0.107	<0.001	(0.09, 0.12)	1 < 2 < 3 < 4
	2.Only IGD	5.77 ± 4.85					
	3.Only SPA	7.54 ± 5.05					
	4.SPA-IGD	9.39 ± 5.72					
Insomnia	1.No SPA-No IGD	6.11 ± 4.04	51.60	0.048	<0.001	(0.04, 0.06)	1 < 2 < 3 (4)
	2.Only IGD	7.18 ± 5.10					
	3.Only SPA	8.24 ± 4.36					
	4.SPA-IGD	8.50 ± 5.32					
Self-control	1.No SPA-No IGD	63.76 ± 10.42	162.58	0.138	<0.001	(0.12, 0.16)	1 (2) > 3 > 4
	2.Only IGD	63.29 ± 11.43					
	3.Only SPA	54.93 ± 11.09					
	4.SPA-IGD	52.37 ± 11.24					
Support utilization	1.No SPA-No IGD	20.93 ± 6.28	22.18	0.021	<0.001	(0.02, 0.03)	1 (2) > 3 > 4
	2.Only IGD	20.88 ± 6.38					
	3.Only SPA	19.40 ± 6.39					
	4.SPA-IGD	18.02 ± 5.88					

### Differences in parenting styles among the four groups

There were significant differences in the scores on all dimensions of parenting styles among the four groups.

*Post-hoc* tests showed that the mother’s care level decreased sequentially among the No SPA-No IGD, Only SPA, and SPA-IGD (Only IGD) groups, while there was no significant difference between the SPA-IGD and Only SPA groups. The father’s care level in the No SPA-No IGD group was the highest among the four groups, but there were no significant differences among the other three groups. The Only-IGD group had the lowest levels of the mother’s encouragement of autonomy and the father’s encouragement of autonomy among the four groups, with no significant differences among the other three groups. The levels of mother’s overprotection and father’s overprotection increased sequentially in the No SPA-No IGD, Only IGD (Only SPA), and SPA-IGD groups, but there were no significant differences between the Only IGD and Only SPA groups (Table 3).

**TABLE 3:** Differences in parenting styles among the four groups (*n* = 3,049).

Parenting styles	Groups	M ± SD	*F*	η*p*^2^	*P*	95% CI	*Post-hoc* test
Mother’s care	1.No SPA-No IGD	21.30 ± 5.93	79.65	0.072	<0.001	(0.06, 0.09)	1 > 3 > 4 (2)
	2.Only IGD	17.28 ± 3.48					
	3.Only SPA	18.89 ± 5.43					
	4.SPA-IGD	17.44 ± 4.62					
Mother’s encouragement of autonomy	1.No SPA-No IGD	10.25 ± 4.28	31.76	0.030	<0.001	(0.02, 0.04)	1 (3) (4) > 2
	2.Only IGD	7.44 ± 4.94					
	3.Only SPA	10.21 ± 4.03					
	4.SPA-IGD	10.26 ± 4.09					
Mother’s overprotection	1.No SPA-No IGD	5.40 ± 3.79	76.58	0.070	<0.001	(0.06, 0.08)	1 < 2 (3) < 4
	2.Only IGD	6.78 ± 4.57					
	3.Only SPA	7.23 ± 4.15					
	4.SPA-IGD	8.77 ± 4.33					
Father’s care	1.No SPA-No IGD	19.64 ± 6.03	31.63	0.030	<0.001	(0.02, 0.04)	1 > 2 (3) (4)
	2.Only IGD	17.03 ± 3.95					
	3.Only SPA	17.91 ± 5.47					
	4.SPA-IGD	17.34 ± 4.85					
Father’s encouragement of autonomy	1.No SPA-No IGD	10.54 ± 4.38	23.44	0.023	<0.001	(0.01, 0.03)	1 (3) (4) > 2
	2.Only IGD	8.05 ± 5.05					
	3.Only SPA	10.46 ± 4.20					
	4.SPA-IGD	10.44 ± 4.08					
Father’s overprotection	1.No SPA-No IGD	4.72 ± 3.94	86.15	0.078	<0.001	(0.06, 0.09)	1 < 2 (3) < 4
	2.Only IGD	7.15 ± 4.72					
	3.Only SPA	6.63 ± 4.59					
	4.SPA-IGD	8.26 ± 4.54					

### Multiple regression analysis of depression, anxiety, and insomnia

Table 4 shows the results of multiple regression analysis. After controlling for gender and age, both depression and anxiety showed negatively correlations with self-control, support utilization, the mother’s care and the father’s encouragement of autonomy, but showed positively relationship with SPA, IGD and the mother’s overprotection. Insomnia showed negatively relationship with self-control and support utilization, but showed positively correlations with SPA and IGD. The result of checking for collinearity of multiple regression analysis of depression, anxiety, and insomnia are detailed in Supplementary Table 3.

**TABLE 4:** Multiple regression analysis on depression, anxiety and insomnia (*n* = 3,049).

Dependent variable	Independent variable	*Coef*	*SE*	*t*	*P*	β	*R* ^2^	*R* ^2^ *_*adj*_*
Depression							0.410	0.407
	Gender	0.814	0.175	4.665	<0.001	0.069		
	Age	0.007	0.055	0.132	0.895	0.002		
	SPA	0.074	0.008	9.669	<0.001	0.165		
	IGD	0.140	0.034	4.111	<0.001	0.065		
	Self-control	–0.194	0.009	–21.856	<0.001	–0.377		
	Support utilization	–0.164	0.014	–11.400	<0.001	–0.176		
	Mother’s care	–0.070	0.021	–3.292	0.001	–0.069		
	Mother’s encouragement of autonomy	0.048	0.030	1.608	0.108	0.035		
	Mother’s overprotection	0.077	0.031	2.475	0.013	0.054		
	Father’s care	0.006	0.019	0.306	0.760	0.006		
	Father’s encouragement of autonomy	–0.086	0.029	–2.948	0.003	–0.064		
	Father’s overprotection	0.039	0.029	1.362	0.173	0.029		
Anxiety							0.325	0.322
	Gender	0.462	0.159	2.909	0.004	0.046		
	Age	–0.047	0.050	–0.939	0.348	–0.015		
	SPA	0.064	0.007	9.128	<0.001	0.166		
	IGD	0.128	0.031	4.124	<0.001	0.070		
	Self-control	–0.146	0.008	–18.166	<0.001	–0.336		
	Support utilization	–0.095	0.013	–7.310	<0.001	–0.121		
	Mother’s care	–0.048	0.019	–2.499	0.012	–0.056		
	Mother’s encouragement of autonomy	–0.008	0.027	–0.312	0.755	–0.007		
	Mother’s overprotection	0.073	0.028	2.568	0.010	0.060		
	Father’s care	0.018	0.018	1.021	0.307	0.021		
	Father’s encouragement of autonomy	–0.056	0.026	–2.097	0.036	–0.049		
	Father’s overprotection	0.040	0.026	1.514	0.130	0.035		
Insomnia							0.181	0.177
	Gender	0.301	0.154	1.960	0.050	0.034		
	Age	–0.192	0.049	–3.932	<0.001	–0.067		
	SPA	0.053	0.007	7.794	<0.001	0.156		
	IGD	0.075	0.030	2.485	0.013	0.047		
	Self-control	–0.093	0.008	–11.910	<0.001	–0.242		
	Support utilization	–0.068	0.013	–5.373	<0.001	–0.098		
	Mother’s care	–0.023	0.019	–1.241	0.215	–0.030		
	Mother’s encouragement of autonomy	–0.014	0.026	–0.518	0.605	–0.013		
	Mother’s overprotection	–0.006	0.027	–0.231	0.817	–0.006		
	Father’s care	–0.012	0.017	–0.685	0.494	–0.015		
	Father’s encouragement of autonomy	–0.017	0.026	–0.673	0.501	–0.017		
	Father’s overprotection	–0.008	0.025	–0.326	0.745	–0.008		

Before being placed in the regression equation, gender was virtualized (male = 0, female = 1).

### Relative weight analysis of depression, anxiety, and insomnia

Table 5 shows the results of relative weight analysis. SPA, self-control and support utilization contributed most to the effects on depression, anxiety and insomnia.

**TABLE 5:** Relative weight analysis on depression, anxiety and insomnia (*n* = 3,049).

Dependent variable	Independent variable	Raw relative weights	Relative weights	*R* ^2^
Depression				0.410
	Gender	0.005	1.337	
	Age	0.001	0.186	
	SPA	0.083	20.20	
	IGD	0.019	4.750	
	Self-control	0.175	42.721	
	Support utilization	0.061	15.010	
	Mother’s care	0.019	4.602	
	Mother’s encouragement of autonomy	0.002	0.602	
	Mother’s overprotection	0.020	4.860	
	Father’s care	0.008	1.935	
	Father’s encouragement of autonomy	0.002	0.414	
	Father’s overprotection	0.014	3.390	
Anxiety				0.325
	Gender	0.003	0.856	
	Age	0.001	0.322	
	SPA	0.074	22.67	
	IGD	0.019	5.970	
	Self-control	0.138	42.55	
	Support utilization	0.036	11.215	
	Mother’s care	0.015	4.517	
	Mother’s encouragement of autonomy	0.002	0.466	
	Mother’s overprotection	0.018	5.630	
	Father’s care	0.005	1.442	
	Father’s encouragement of autonomy	0.001	0.378	
	Father’s overprotection	0.013	3.984	
Insomnia				0.181
	Gender	0.002	0.943	
	Age	0.006	3.526	
	SPA	0.049	26.98	
	IGD	0.009	5.139	
	Self-control	0.076	42.138	
	Support utilization	0.023	12.499	
	Mother’s care	0.006	3.186	
	Mother’s encouragement of autonomy	0.007	0.392	
	Mother’s overprotection	0.003	1.721	
	Father’s care	0.004	2.075	
	Father’s encouragement of autonomy	0.001	0.359	
	Father’s overprotection	0.002	1.043	

Before being placed in the regression equation, gender was virtualized (male = 0, female = 1).

### Multiple regression analysis of SPA and IGD

Table 6 shows the results of another multiple regression analysis. After controlling for gender and age, SPA showed negatively correlations with self-control, support utilization and the mother’s care but showed positively relationship with parental overprotection. IGD showed negatively relationship with self-control, the mother’s care and parental encouragement of autonomy but showed positively correlations with the father’s overprotection. The result of checking for collinearity of multiple regression analysis of SPA and IGD are detailed in Supplementary Table 4.

**TABLE 6:** Multiple regression analysis on SPA and IGD (*n* = 3,049).

Dependent variable	Independent variable	*Coef*	*SE*	*t*	*P*	β	*R* ^2^	*R* ^2^ *_*adj*_*
SPA							0.293	0.291
	Gender	–1.081	0.408	–2.648	0.008	–0.041		
	Age	–0.197	0.135	–1.466	0.143	–0.023		
	Self-control	–0.511	0.019	–26.340	<0.001	–0.447		
	Support utilization	–0.069	0.035	–1.967	0.049	–0.033		
	Mother’s care	–0.116	0.052	–2.253	0.024	–0.051		
	Mother’s encouragement of autonomy	0.080	0.072	1.110	0.267	0.026		
	Mother’s overprotection	0.230	0.076	3.033	0.002	0.072		
	Father’s care	–0.009	0.047	–0.200	0.842	–0.004		
	Father’s encouragement of autonomy	0.024	0.071	0.343	0.731	0.008		
	Father’s overprotection	0.263	0.070	3.753	<0.001	0.087		
IGD							0.187	0.184
	Gender	–1.464	0.091	–16.003	<0.001	–0.266		
	Age	0.045	0.030	1.491	0.136	0.025		
	Self-control	–0.049	0.004	–11.212	<0.001	–0.204		
	Support utilization	0.001	0.008	0.184	0.854	0.003		
	Mother’s care	–0.045	0.012	–3.894	<0.001	–0.095		
	Mother’s encouragement of autonomy	–0.040	0.016	–2.451	0.014	–0.063		
	Mother’s overprotection	0.009	0.017	0.542	0.588	0.014		
	Father’s care	0.004	0.011	0.374	0.709	0.008		
	Father’s encouragement of autonomy	–0.039	0.016	–2.447	0.014	–0.063		
	Father’s overprotection	0.086	0.016	5.444	<0.001	0.135		

Before being placed in the regression equation, gender was virtualized (male = 0, female = 1).

### A test of the multiple mediating roles of support utilization and self-control between parenting styles and SPA or IGD

Table 7 shows the path model results. When SPA was set as dependent variable, model fit index was good (χ^2^/df = 13.12, RMESA = 0.063, CFI = 0.953, TLI = 0.858). When IGD was set as dependent variable, model fit index was good as well (χ^2^/df = 13.11, RMESA = 0.062, CFI = 0.940, TLI = 0.821).

**TABLE 7:** Effects of support utilization and self-control between parenting styles and SPA and IGD (*n* = 3,049).

	Total effect			Indirect effect (self-control)			Indirect effect (support utilization)			Sequential indirect effect (support utilization→self-control)			Direct effect		
					
	β	*P*	95% CI	β	*P*	95% CI	β	*P*	95% CI	β	*P*	95% CI	β	*P*	95% CI
**SPA**															
MC	–0.291	<0.001	[–0.406 –0.175]	–0.109	<0.001	[–0.164 –0.054]	–0.018	0.070	[–0.038 0.001]	–0.053	<0.001	[–0.070 –0.037]	–0.110	0.031	[–0.211 –0.010]
MEA	0.258	0.007	[0.072 0.443]	0.156	<0.001	[0.074 0.237]	0.006	0.240	[–0.004 0.016]	0.017	0.085	[–0.002 0.037]	0.079	0.334	[–0.081 0.238]
MO	0.476	<0.001	[0.279 0.673]	0.210	<0.001	[0.122 0.298.]	0.008	0.158	[–0.003 0.020]	0.024	0.021	[0.004 0.045]	0.233	0.006	[0.068 0.399]
FC	–0.171	0.001	[–0.275 –0.068]	–0.109	<0.001	[–0.158 –0.060]	–0.014	0.071	[–0.028 0.001]	–0.040	<0.001	[–0.054 –0.025]	–0.009	0.855	[–0.102. 0.085]
FEA	0.226	0.011	[0.052 0.399]	0.131	0.001	[0.054 0.208]	0.017	0.078	[–0.002 0.036]	0.050	<0.001	[0.029 0.071]	0.029	0.709	[–0.121 0.178]
FO	0.219	0.018	[0.038. 0.401]	–0.012	0.757	[–0.090 0.066]	–0.004	0.323	[–0.013 0.004]	–0.012	0.196	[–0.031 0.006]	0.248	0.002	[0.094 0.402]
**IGD**															
MC	–0.061	<0.001	[–0.082 –0.040]	–0.010	<0.001	[–0.016 –0.005]	0.001	0.738	[–0.003 0.004]	–0.005	<0.001	[–0.007 –0.003]	–0.046	<0.001	[–0.067 –0.026]
MEA	–0.023	0.153	[–0.055 0.009]	0.015	<0.001	[0.007 0.023]	<0.001	0.773	[–0.002 0.001]	0.002	0.086	[–0.001 0.003]	–0.039	0.014	[–0.071 –0.008]
MO	0.030	0.082	[–0.004 0.064]	0.020	<0.001	[0.011 0.029]	<0.001	0.756	[–0.002 0.002]	0.002	0.023	[0.001 0.004]	0.008	0.616	[–0.025 0.042]
FC	–0.010	0.298	[–0.028 0.009]	–0.010	<0.001	[–0.015 –0.005]	0.001	0.741	[–0.002 0.003]	–0.004	<0.001	[–0.005 –0.002]	0.004	0.677	[–0.014 0.022]
FEA	–0.023	0.128	[–0.054 0.007]	0.012	0.002	[0.005 0.020]	–0.001	0.742	[–0.004 0.003]	0.005	<0.001	[0.003 0.007]	–0.040	0.009	[–0.070 –0.010]
FO	0.087	<0.001	[0.056 0.118]	–0.001	0.758	[–0.009 0.006]	<0.001	0.792	[–0.001 0.001]	–0.001	0.200	[–0.003 0.001]	0.089	<0.001	[0.058 0.120]

The effects were tested by using the bootstrap method with 5,000 resamples. MC, mother’s care; MEA, mother’s encouragement of autonomy; MO, mother’s overprotection; FC, father’s care; FEA, father’s encouragement of autonomy; FO, father’s overprotection.

**FIGURE 1:**
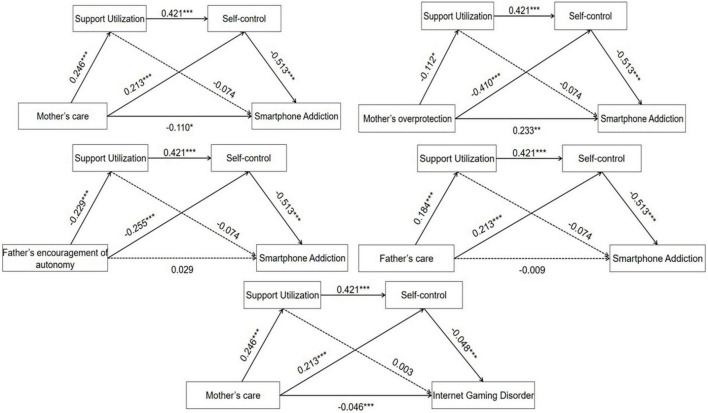
Path models of parenting styles dimensions which had significant sequential indirect effects through multiple mediation of support utilization and self-control on smartphone addiction and Internet gaming disorder. Dotted lines indicate non-significant relations. *p < 0.05, **p < 0.01, ***p < 0.001.

According to models, all parenting styles had no significant partially indirect effect on SPA or IGD through support utilization. In addition to the mother’s encouragement of autonomy and father’s overprotection, parental care, mother’s overprotection, and father’s encouragement of autonomy had a significant sequential indirect effect on SPA through the multiple mediation of support utilization and self-control. However, only the mother’s care had a significant sequential indirect effect on IGD through the multiple mediation. Specific path coefficients are detailed in Figure 1.

## Discussion

By investigating depression, anxiety, insomnia as well as other psychological characteristics as proxies for adolescents’ mental health and their past experienced parenting styles, the present study found that although both SPA and IGD threatened adolescents’ mental health, adolescents with only SPA showed worse mental health status compared to adolescents with only IGD. Moreover, high levels of positive parenting styles and low levels of negative parenting styles were beneficial to adolescents’ mental health overall, but it was less valid for adolescents with SPA. The impacts of parenting styles on adolescents’ mental health were not direct, but rather indirectly manifested through the intrinsic psychological mechanisms of support utilization and self-control that influence high-risk Internet use behaviors. Importantly, encouraging autonomy showed negative and convergent effects with caring on adolescents’ IGD, but reveled positive and divergent effects with caring on adolescents’ SPA.

In this study, compared to adolescents in group no SPA-no IGD, adolescents with SPA or IGD had higher levels of depression, anxiety, and insomnia, which is consistent with previous studies (Demirci et al., 2015; Kircaburun et al., 2019). In contrast, adolescents with SPA and IGD had lower levels of self-control and support utilization and that is consistent with prior studies showing that abilities of self-control and support utilization were protective factors against high-risk Internet use behaviors (Spada, 2014; Kim et al., 2016; Wu X. S. et al., 2016; Mo et al., 2018). The IGD-SPA comorbidity group had the highest levels of depression, anxiety, and insomnia. A previous study showed that using smartphones for gaming increased the time length or frequency of smartphone usage and further increased the risk of smartphone addiction (van Deursen et al., 2015). Meanwhile, IGD may also reinforce SPA behavior (Chou and Chou, 2019). Thus, SPA and IGD comorbidity may have critically negative impacts on adolescents’ mental health (Chang et al., 2019; Elhai et al., 2019). We also found that the only SPA group had significantly higher levels of depression, anxiety, and insomnia degrees than the only IGD group. Adolescents are more susceptible to peer influence (Steinberg and Monahan, 2007; Somerville, 2013) and pursue peer relationships even more than family relationships (Lee and Kim, 2018). Based on the finding that the usage of social software (QQ & WeChat) in the Only SPA group had the highest percentage among the four groups (Supplementary Table 1), we suggest that the other Internet addiction behaviors (e.g., virtual socialization) may cause adolescents to spent greater time on the Internet than gaming and result in worse mental health states (Kuss et al., 2013; Montag et al., 2015b; Liu et al., 2018; Sun et al., 2019). The adverse effects of IGD on mental health, well-being, and daily functioning have been widely recognized (Sarda et al., 2016). Although a consensus has been reached that SPA is harmful to adolescents’ mental health, people may underestimate the actual damage of SPA to adolescents when they are not using smartphones for gaming. However, our study revealed that the mental health of adolescents with SPA but without IGD might be more vulnerable to depression, anxiety, and insomnia.

In this study, adolescents without SPA and IGD not only had lowest levels of depression, anxiety, and insomnia, but also associated with highest levels of the caring parenting styles, encouraging autonomy parenting styles and lowest levels of the overprotection parenting styles, consistent with previous finding that positive parenting styles can reduce the incidence of depression in adolescents (Collishaw et al., 2016). However, the only SPA group had a higher level of mother’s care than the only IGD group but exhibited a worse mental health state. Further relative weight analysis showed that adolescents’ mental health indexes mainly related to SPA, self-control and support utilization. This result indicated that effects of parenting styles on adolescents’ mental health may need to be externalized indirectly by impacting adolescents’ high-risk Internet use behaviors. Therefore, positive parenting styles should be used as a pre-emptive strategy to prevent adolescents from developing smartphone addictive behaviors, not as a remedy for improving the mental health of adolescents with SPA. Thus, simply emphasizing the defensive effects of positive family parenting styles alone would not achieve the desired interventions on mental health of adolescents with SPA.

In the present study, high levels of the caring parenting styles and low levels of the overprotection parenting styles might be protective factors for adolescents’ SPA and IGD behaviors, consistent with previous findings that positive parenting styles negatively affect SPA and IGD, while negative parenting styles positively affect both of them (Deng et al., 2015; Lian et al., 2016). By constructing a pathway model, we found that parental care and the mother’s overprotection indirectly influenced SPA through the multiple mediation effects of support utilization and self-control, and the mother’s care indirectly affected IGD through support utilization and self-control. This multiple mediation effects of support utilization and self-control highlighted the importance of family parenting styles in altering the self-control resources of adolescents. The positive effects of parental care on self-control were in line with the finding that children have higher levels of self-control when parents convey more warmth to their children (Crosswhite and Kerpelman, 2012). The negative effect of mother’s overprotection on self-control was also in line with the finding that children with less self-control had limited effective parenting styles (Ng-Knight et al., 2016). In particular, negative parenting styles reduce adolescents’ ability to utilize social support, and that may cause adolescents to feel low levels of social support (Karaer and Akdemir, 2019) and self-control (Wu et al., 2017). When faced with negative parenting styles, adolescents do not necessarily have the opportunity to receive vicarious compensation or emotional buffers from a social support system to help them resist temptations from undesirable behaviors, such as online gaming or indulging in smartphone use.

Notably, the parenting style of encouraging autonomy showed surprising divergent effects on adolescents’ IGD and SPA. The father’s encouragement of autonomy positively influenced SPA in adolescents. However, parental encouragement of autonomy negatively predicted IGD in adolescents. Rossé (2012) argued that when parents hinder adolescents from seeking independence to reconstruct their self-identity, adolescents may avoid communication with their parents by becoming addicted to the Internet. Giving adolescents freedom may help them refine their self-identity and build closer relationships with their parents, reducing the risk of IGD (Deng et al., 2013). However, smartphones, as a media platform enabling various types of Internet behaviors, are likely to make individuals extremely dependent (Hoffner et al., 2015; De-Sola Gutiérrez et al., 2016). Giving adolescents freedom may push them to be prone to suffer in SPA. Especially, the father’s encouragement of autonomy indirectly influenced SPA through the negative multiple mediation effects of support utilization and self-control. Therefore, emphasis on independence too early may trigger SPA in adolescents who are still in developmental stages, regardless of what purposes they use their smartphones for. More importantly, based on the outstanding contribution to adolescent’s mental health of self-control, the negative impact of encouraging autonomy on self-control showed that the autonomy-encouraging parenting style had a similar effect with the overprotection parenting style not only in strengthening SPA behaviors in adolescents but also in damaging mental health of adolescents. Previous researches have considered encouraging autonomy and overprotection as two antagonistic parenting styles (Costa et al., 2016; Inguglia et al., 2018). However, when synergizing with low parental monitoring, adolescents exhibit a higher degree of internal psychological issues with autonomy-encouraging or overprotective parenting styles (Rodriguez-Meirinhos et al., 2020). Therefore, this study implied that both encouraging autonomy and overprotection parenting styles are detrimental to adolescents with SPA, and parental monitoring is necessary for controlling adolescents’ smartphone use behaviors.

Additionally, lacking self-control can aggravate different behavioral problems and addictions (Dvorak et al., 2011; Oezdemir et al., 2014). SPA may have different addiction motives than IGD (Noë et al., 2019; Nie et al., 2020). Therefore, the psychological satisfaction sought by adolescents during SPA behaviors may not be the same as that sought in Internet games. Thus, the divergent effect of the encouraging autonomy parenting style on IGD and SPA may be due to self-control playing different roles in the maintenance and formation process of various types of Internet addiction.

## Limitation

Although the questionnaires used in this study have been shown to have good reliability in previous studies, it should be noted that Cronbach’ alpha coefficients for MPAI, GAD, PHQ and the support utilization subscale were slightly above the recommended range (0.90) (Streiner, 2003) in this study. There may be over-high correlations among the items within each of these four questionnaires, meaning that participants’ SPA, anxiety, depression and support utilization may only be measured from a narrow profile. Moreover, the participants for this study were junior and senior high school adolescents and the mental health status of adolescent is not homeostatic. Adolescents are in a critical developmental period with active emotional-behavioral function and risk for psychopathology, their mental health status can fluctuate (Costello et al., 2003). For example, depression in adolescents may vary with development (Prenoveau et al., 2011). Future researchers could use longitudinal design to increase the stability of results.

## Conclusion

In conclusion, this study found that adolescents with SPA and IGD comorbidity had the highest levels of depression, anxiety, and insomnia levels. Despite having a more positive parenting style, adolescents with SPA but no IGD showed more severe mental health problems than adolescents with only IGD. Moreover, the autonomy-encouraging parenting style protected against IGD but strengthened SPA in adolescents through the multiple mediation effects of support utilization and self-control. This study suggests that parents should adopt different supervisory strategies to deal with different types of Internet addiction.

## Data availability statement

The raw data supporting the conclusions of this article will be made available by the authors, without undue reservation.

## Ethics statement

The studies involving human participants were reviewed and approved by Ethics Committee of the School of Education, Hunan University of Science and Technology, China. Written informed consent to participate in this study was provided by the participants’ legal guardian/next of kin.

## Author contributions

Z-KL and L-JS developed the study concept and designed the study. Z-KL analyzed and interpreted the data. Z-KL, L-JS, and X-LC prepared the manuscript. L-JS provided critical revisions. All authors performed data collection, had full access to all data in the study and take responsibility for the integrity of the data and the accuracy of the data analysis, and approved the final version of the manuscript for submission.

## Footnotes

1
http://wjx.cn
