# Supplementary material for: Smartphone addiction is more harmful to adolescents than Internet gaming disorder: Divergence in the impact of parenting styles
Source: Front Psychol. 2022 Dec 14;13:1044190. doi: 10.3389/fpsyg.2022.1044190 (PMC9796998; doi:10.3389/fpsyg.2022.1044190)
Supplement: Supplementary file 1 [file Data_Sheet_1.docx]

Supplementary Material

S2 ESTIMATION EQUATION

***Y_i_*=*a*+β_1_*X_1i_+*β_2_*X_2i_+***…***+*β_j_*X_ji_+*β_1'_*X^'^_1_+*β_2'_*X*^'^*_2_* (1)**

Equation 1 was the estimated model of multiple regression analysis on depression, anxiety and insomnia. Alpha denotes the intercept of regression equation and beta denotes the regression coefficients of independent variables; *i* was 1, 2, and 3. The dependent variables *Y_i_* were depression (*Y_1_*), anxiety (*Y_2_*), and insomnia (*Y_3_*) respectively. And j was 1 to 10. The independent variables *X_j_* were SPA (*X_1_*), IGD (*X_2_*), self-control (*X_3_*), support utilization (*X_4_*), mother's care (*X_5_*), mother's encouragement of autonomy (*X_6_*), mother's overprotection (*X_7_*), father's care (*X_8_*), father's encouragement of autonomy (*X_9_*), father's overprotection (*X_10_*). X' were gender (*X^'^_1_*) and age (*X^'^_2_*).

***ℇ_ij_* = λ^2^*_ij1_β^2^*_1i_ *+* λ^2^*_ij2_β^2^*_2i_ *+* …+ λ^2^*_ijk_β^2^*_ki_  (2)**

Equation 2 was the estimation model for Relative weight analysis on depression, anxiety and insomnia. λ^2^*_ijk_β^2^*_ki_ can be interpreted as the proportion of variance in *Y_i_* that is associated with *X_j_* through its relationship with *Z_kXj._* The summing of *ℇ_ij_* was the total proportion of variance in *Y_i_* that was associated with *X_j._* And *i* was 1, 2, and 3. The dependent variables *Y_i_* were depression (*Y_1_*), anxiety (*Y_2_*), and insomnia (*Y_3_*) respectively. And j was 1 to 12. The independent variables *X_j_* were gender (*X_1_*), age (*X_2_*), SPA (*X_3_*), IGD (*X_4_*), self-control (*X_5_*), support utilization (*X_6_*), mother's care (*X_7_*), mother's encouragement of autonomy (*X_8_*), mother's overprotection (*X_9_*), father's care (*X_10_*), father's encouragement of autonomy (*X_11_*), father's overprotection (*X_12_*).

***Y_k_*=*a^‘^*+β_1_*X_1k_+*β_2_*X_2k_+***…***+*β*_h_X_hk_+*β_1'_*X^'^_1_+*β_2'_*X*^'^*_2_* (3)**

Equation 3 was the estimated model of multiple regression analysis on SPA and IGD. Alpha^'^ denotes the intercept of regression equation and beta denotes the regression coefficients of independent variables; *k* was 1 and 2. The dependent variables *Y_k_* were SPA (*Y_1_*) and IGD (*Y_2_*) respectively. And *h* was 1 to 10. The independent variables *X_h_* were self-control (*X_1_*), support utilization (*X_2_*), mother's care (*X_3_*), mother's encouragement of autonomy (*X_4_*), mother's overprotection (*X_5_*), father's care (*X_6_*), father's encouragement of autonomy (*X_7_*), father's overprotection (*X_8_*). X' were gender (*X^'^_1_*) and age (*X^'^_2_*).

***Y_i_ = c_i_X_ji_ + ε_1i_* (4)**

***M_1_ = a_1j_X_j_ + ε_2j_* (5)**

***M_2_ = a_2j_X_j_ + d_21j_M_1_ + ε_3j_* (6)**

***Y_i_ = c^'^_i_X_ji_ + b_1i_M_1_ + b_2i_M_2_ + ε_4i_* (7)**

***Y_i_ =* (*c^'^_i_* + *a_1j_b_1i_* + *a_2j_b_2i_ + a_1j_d_21j_b_2i_*)*X_j_ +* (*b_1i_*+ *d_21j_b_2i_*)*ε_2j_ + b_2i_ε_3j_ + ε_4i_* (8)**

***Indirect effect of X_j_ on Y_i_ through M_1_ only = a_1j_b_1i_***

***Indirect effect of X_j_ on Y_i_ through M_2_ only = a_2j_b_2i_***

***Indirect effect of X_j_ on Y_i_ through M_1_ and M_2_ in serial = a_1j_d_21j_b_2i_***

***Direct effect of X_j_ on Y_i_ = c^'^_i_***

***Total effect of X_j_ on Y_i_ = c_i_***

Equations 4 - 8 were the estimated models of multiple mediation analysis on SPA and IGD. The regression coefficient *c_i_* represents the effect of the independent variable *X_j_* on the dependent variable *Y_i_*_._ The regression coefficient *a_1j_* and *a_2j_* indicated the effect of the independent variable *X_j_* on the mediating variables *M_1_* and *M_2_*, respectively. The regression coefficient *d_21j_* indicated the effect of *M_1_* on *M_2_* when independent variable was *X_j_* . The regression coefficients *b_1i_* and *b_2i_* were the effects of *M_1_* and *M_2_* on the dependent variable *Y_i_*_._ after controlling for the effect of the independent variable *X_j_*. The regression coefficients *c^'^_i_* was the effect of *X_j_* on *Y_i_* after controlling for the effects of *M_1_* and *M_2_*. And *ε_1i_*, *ε_2j_*, *ε_3j_*, and *ε_4i_* represented residuals, which were assumed to be normally distributed and independent of each other. And *i* was 1 and 2. The dependent variables *Y_i_*_._ SPA (*Y_1_*) and IGD (*Y_2_*) respectively. And j was 1 to 6. The independent variables *X_j_* were mother's care (*X_1_*), mother's encouragement of autonomy (*X_2_*), mother's overprotection (*X_3_*), father's care (*X_4_*), father's encouragement of autonomy (*X_5_*), father's overprotection (*X_6_*).
