# Supplementary material for: Smartphone addiction is more harmful to adolescents than Internet gaming disorder: Divergence in the impact of parenting styles
Source: Front Psychol. 2022 Dec 14;13:1044190. doi: 10.3389/fpsyg.2022.1044190 (PMC9796998; doi:10.3389/fpsyg.2022.1044190)
Supplement: Supplementary file 2 [file Table_1.docx]

Supplementary Material

Table S1. The percentages of smartphone usage among participants in the four groups

| Group  Percentage  Usage | No SPA–No IGD  (*n* = 2002) | Only IGD  (*n* = 240) | Only SPA  (*n* = 550) | SPA–IGD  (*n* = 257) |
| --- | --- | --- | --- | --- |
| Phone calls (1361) | 911 (45.50%) | 109 (45.40%) | 234 (42.50%) | 107 (41.60%) |
| Messages (747) | 483 (24.10%) | 59 (24.60%) | 138 (25.10%) | 67 (26.10%) |
| Shopping (1219) | 765 (38.20%) | 92 (38.30%) | 262 (47.60%) | 100 (38.90%) |
| Gaming (1568) | 931 (46.50%) | 142 (59.20%) | 310 (56.40%) | 185 (72.0%) |
| Social network (2314) | 1507 (75.20%) | 171 (71.30%) | 451 (82.0%) | 187 (75.90%) |
| Listening to music (2102) | 1404 (70.10%) | 147 (61.30%) | 387(70.40%) | 164 (63.80%) |
| Learning (1460) | 1055 (52.70%) | 80 (33.30%) | 237 (43.10%) | 88 (34.20%) |
| TikTok (1750) | 1072 (53.50%) | 148 (61.70%) | 368 (66.90%) | 162 (63.0%) |
| Others (791) | 536 (26.80%) | 46 (19.20%) | 140 (25.50%) | 69 (26.80) |

Note: The number shown in parentheses is the total number of participants using smartphones for that purpose in the overall sample, and the number under each group is the specific number of participants using smartphones for that purpose in that group.
