# Supplementary material for: Smartphone addiction is more harmful to adolescents than Internet gaming disorder: Divergence in the impact of parenting styles
Source: Front Psychol. 2022 Dec 14;13:1044190. doi: 10.3389/fpsyg.2022.1044190 (PMC9796998; doi:10.3389/fpsyg.2022.1044190)
Supplement: Supplementary file 3 [file Table_2.docx]

Supplementary Material

Table S2. Check for collinearity of multiple regression analysis on depression, anxiety and insomnia

| **Dependent variable** | **Independent variable** | **VIF** | **Mean VIF** |
| --- | --- | --- | --- |
| **depression**  **anxiety**  **insomnia** |  |  | 1.79 |
|  | gender | 1.123 |  |
|  | age | 1.077 |  |
|  | SPA | 1.493 |  |
|  | IGD | 1.299 |  |
|  | Self-control | 1.534 |  |
|  | support utilization | 1.222 |  |
|  | mother's care | 2.230 |  |
|  | mother's encouragement of autonomy | 2.453 |  |
|  | mother's overprotection | 2.431 |  |
|  | father's care | 1.847 |  |
|  | father's encouragement of autonomy | 2.450 |  |
|  | father's overprotection | 2.339 |  |
