# Supplementary material for: Smartphone addiction is more harmful to adolescents than Internet gaming disorder: Divergence in the impact of parenting styles
Source: Front Psychol. 2022 Dec 14;13:1044190. doi: 10.3389/fpsyg.2022.1044190 (PMC9796998; doi:10.3389/fpsyg.2022.1044190)
Supplement: Supplementary file 4 [file Table_3.docx]

Supplementary Material

Table S3. Check for collinearity of multiple regression analysis on SPA and IGD

| **Dependent variable** | **Independent variable** | **VIF** | **Mean VIF** |
| --- | --- | --- | --- |
| **SPA**  **IGD** |  |  | 1.83 |
|  | gender | 1.035 |  |
|  | age | 1.075 |  |
|  | self-control | 1.239 |  |
|  | support utilization | 1.220 |  |
|  | mother's care | 2.218 |  |
|  | mother's encouragement of autonomy | 2.446 |  |
|  | mother's overprotection | 2.424 |  |
|  | father's care | 1.847 |  |
|  | father's encouragement of autonomy | 2.445 |  |
|  | father's overprotection | 2.311 |  |
